# PET imaging of [^11^C]PBR28 in Parkinson’s disease patients does not indicate increased binding to TSPO despite reduced dopamine transporter binding

**DOI:** 10.1007/s00259-018-4161-6

**Published:** 2018-10-01

**Authors:** Katarina Varnäs, Zsolt Cselényi, Aurelija Jucaite, Christer Halldin, Per Svenningsson, Lars Farde, Andrea Varrone

**Affiliations:** 1 Department of Clinical Neuroscience, Center for Psychiatry Research, Karolinska Institutet and Stockholm County Council, R5:02 Karolinska University Hospital, SE-17176 Stockholm, Sweden; 2 PET Science Centre, Precision Medicine and Genomics, IMED Biotech Unit, AstraZeneca, Karolinska Institutet, Stockholm, Sweden; 3 Department of Clinical Neuroscience, Translational Neuropharmacology, Center for Molecular Medicine, Karolinska Institutet, Stockholm, Sweden

**Keywords:** PET imaging, Parkinson’s disease, 18 kDa translocator protein, Dopamine transporter

## Abstract

**Purpose:**

To examine the hypothesis that cerebral binding to the 18 kDa translocator protein (TSPO), a marker of microglia activation, is elevated in Parkinson’s disease (PD), and to assess the relationship between brain TSPO binding and dopaminergic pathology in PD.

**Methods:**

The radioligand [^11^C]PBR28 was used for quantitative assessment of brain TSPO in 16 control subjects and 16 PD patients. To analyse the relationship between dopaminergic pathology and brain TSPO binding, PET studies of the dopamine transporter (DAT) were undertaken in PD patients using the DAT radioligand [^18^F]FE-PE2I. The total distribution volume of [^11^C]PBR28 was quantified in nigrostriatal regions, limbic cortices and thalamus, and the binding potential of [^18^F]FE-PE2I was quantified in nigrostriatal regions.

**Results:**

Based on genotype analysis of the TSPO rs6971 polymorphism, 16 subjects (8 control subjects and 8 PD patients) were identified as high-affinity binders, and the remaining subjects were identified as mixed-affinity binders. A two-way ANOVA showed a strong main effect of TSPO genotype on the cerebral binding of [^11^C]PBR28, whereas no statistically significant main effect of diagnostic group, or a group by genotype interaction was found for any of the regions analysed. [^18^F]FE-PE2I PET studies in patients indicated a marked reduction in nigrostriatal binding to DAT. However, no correlations between the binding parameters were found for [^11^C]PBR28 and [^18^F]FE-PE2I.

**Conclusion:**

The findings do not support the hypothesis of elevated cerebral TSPO binding or a relationship between TSPO binding and dopaminergic pathology in PD.

**Electronic supplementary material:**

The online version of this article (10.1007/s00259-018-4161-6) contains supplementary material, which is available to authorized users.

## Introduction

Parkinson’s disease (PD) is a neurological condition characterized by degeneration of dopaminergic nigrostriatal neurons, and by the presence of cytoplasmic protein deposits in the affected neuronal pathways [1]. A role for inflammatory processes in PD is supported by evidence from studies of activated microglia [2, 3] and inflammatory cytokines [4] in post-mortem brain tissue from PD patients. However, the relationship between inflammation and the neurodegenerative and behavioural features of PD remains poorly understood.

The 18 kDa translocator protein (TSPO), is known to be expressed in microglia cells, and can be quantified using PET and TSPO radioligands, and is thus a putative biomarker of neuroinflammation in vivo [5]. Among the TSPO PET radioligands developed, [^11^C]PK11195 has been the most widely used in applied studies [6]. However, [^11^C]PK11195 shows low specific binding to TSPO, which limits its use in the assessment of brain microglia in vivo. For this reason, several new TSPO radioligands have been developed with improved signal to noise ratio, including [^11^C]PBR28, [^11^C]DPA713, [^18^F]FEPPA, and [^11^C]DAA1106 [6].

Previous PET studies using [^11^C]PK11195 as a marker of activated microglia have provided support for elevated brain TSPO in PD patients [7–10]. However, these findings have not been consistently confirmed [11]. Two studies combining PET measurement of TSPO and markers of dopaminergic neurodegeneration in the same patients have yielded contrasting findings regarding the relationship between these measures [7, 8]. Moreover, findings in subsequent studies using second generation radioligands have been inconclusive. Whereas elevated TSPO binding was found in a study using the radioligand [^11^C]DPA713 [12], this finding was not supported by investigations using the radioligand [^18^F]FEPPA for assessment of brain TSPO binding in PD patients [13, 14]. The inconclusive findings warrant further study of TSPO in PD using second generation PET radioligands and assessment of the relationship between TSPO binding and dopaminergic pathology in PD.

In the present investigation, the second generation radioligand [^11^C]PBR28 was used for quantitative evaluation of brain TSPO in 16 control subjects and 16 PD patients. To analyse the relationship between brain TSPO binding and dopaminergic pathology, PET measurements of the dopamine transporter (DAT) were undertaken using the radioligand [^18^F]FE-PE2I.

## Materials and methods

### Subjects and study design

The study was approved by the Research Ethics Committee in Stockholm, Sweden, and the Radiation Safety Committee of Karolinska University Hospital, Stockholm, and was performed in accordance with the current amendments of the Declaration of Helsinki and International Conference on Harmonization/ Good Clinical Practice guidelines. Written informed consent was obtained from all participants. The study included 16 control subjects and 16 patients diagnosed with PD. Demographic and clinical information is presented in Table 1.

**Table 1:** Demographic and clinical information

	Control subjects	PD patients
Number of subjects (female/male)	16 (1/15)	16 (1/15)
TSPO genotype (HAB/MAB)^a^	8/8	8/8
Age (years), mean (range)	63 (56–72)	64 (55–73)
Motor UPDRS (part III) score, mean (range)	–	17 (6–29)
Dopamine replacement therapy		
Carbidopa/levodopa	–	1
Dopamine agonist	–	1
Carbidopa/levodopa and dopamine agonist	–	4
Various combinations^b^	–	8
Drug-naive	–	2
[^11^C]PBR28 radiochemistry		
Injected radioactivity (MBq), mean (range)	408 (252–517)	403 (338–438)
Molar radioactivity (GBq/μmol), mean (range)	218 (109–341)	377 (95.0–700)
Injected mass (μg), mean (range)	0.73 (0.35–1.5)	0.52 (0.20–1.5)

*UPDRS* Unified Parkinson’s Disease Rating Scale

^a^rs6971 polymorphism; high-affinity and mixed-affinity binders

^b^Including monoamine oxidase or catechol-O-methyl-transferase inhibitors

The study is an extension of a previous investigation assessing the effect of the myeloperoxidase inhibitor AZD3241 on TSPO binding in PD patients [15]. Patients were examined during the period May to October 2012 within this previous study supported by AstraZeneca. In the present extension, matched control subjects were examined between March 2015 and February 2016. Patients fulfilled the modified UK Parkinson’s Disease Society Brain Bank criteria for idiopathic PD [16]. Inclusion and exclusion criteria have previously been described [15]. The clinical evaluation of patients included collection of demographic and general clinical information, a standardized neurological examination, and assessment of disease severity and motor signs using the Unified Parkinson’s Disease Rating Scale (UPDRS) [17]. Genotyping for TSPO polymorphism [18] to assess the affinity status of [^11^C]PBR28 was performed in accordance with previously described procedures [15, 19], using the TaqMan® assays (Applied Biosystems) and detection via ABI 7900HT SDS (Applied Biosystems).

All subjects underwent PET measurements with the TSPO radioligand [^11^C]PBR28. In addition, for confirmation of nigrostriatal pathology, PD patients were examined using the DAT radioligand [^18^F]FE-PE2I. [^18^F]FE-PE2I binding was analysed by visual assessment of striatal DAT loss, and criteria for inclusion were the presence of asymmetrical decrease in striatal [^18^F]FE-PE2I binding according to the criteria reported in the literature [20, 21].

### Radiochemistry

[^11^C]PBR28 was prepared from its corresponding desmethyl-PBR28 precursor (PharmaSynth AS, Tartu, Estonia), essentially as described elsewhere [22]. [^18^F]FE-PE2I was prepared as previously described [23] from its corresponding tosyl precursor, TsOE-PE2I (PharmaSynth AS). Injected radioactivity, molar radioactivity and injected mass of [^11^C]PBR28 are presented in Table 1. For [^18^F]FE-PE2I the injected radioactivity was 158–195 MBq. The molar radioactivity at the time of injection was 65–433 GBq/μmol, corresponding to an injected mass of 0.20–1.3 μg.

### Imaging procedures

Prior to PET measurements, two anatomical brain MRI examinations were performed in each subject. The first (T2-weighted) images were used for clinical evaluation and exclusion of pathology, and were evaluated by a clinical neuroradiologist. The second (T1-weighted) images were used for coregistration with the PET images and spatial normalization. Images were acquired using a 1.5-T Siemens Avanto system in patients and a 3-T General Electric Discovery MR750 (GE, Milwaukee, WI, USA) system in control subjects. The T1-weighted MR images were reoriented according to the line defined by the anterior and posterior commissures, resampled and cropped to generate a 220 × 220 × 170 matrix with 1 mm^3^ voxels. The images were segmented into grey and white matter, and CSF using SPM8 software (Wellcome Department of Cognitive Neurology, UK) and coregistered with PET images.

An individual plaster helmet was made for each subject and used with a head fixation system to minimize head movement. A cannula was inserted into the left or right cubital vein. The radioligand was dissolved in sterile physiological phosphate buffer (pH 7.4) and injected as a bolus over 10 s. The cannula was then immediately flushed with 10 ml saline.

PET data were acquired with a high-resolution research tomograph (HRRT; Siemens/CTI) over 72 min for [^11^C]PBR28 examinations in control subjects and 93 min for [^11^C]PBR28 and [^18^F]FE-PE2I examinations in patients. The data acquisition protocol was shortened in control subjects based on previous experience indicating that 60 min data acquisition is sufficient for quantification of [^11^C]PBR28 binding to the TSPO [19]. List-mode data were binned and reconstructed as previously described [24].

### Blood radioactivity measurements and arterial plasma input function for [^11^C]PBR28

A catheter was inserted into the radial artery and arterial blood was collected continuously during the first 10 min using an automated blood sampling system (ABSS; Allogg AB, Sweden). In addition, arterial blood samples (2–4 ml) were drawn manually at approximately 2, 4, 6, 8, 10, 15, 20, 25, 30, 40, 50, 70 and 90 min after radioligand injection in patients and 2, 4, 6, 8, 10, 15, 20, 25, 30, 40, 50, 60 and 72 min after radioligand injection in control subjects. The blood sampling protocol in control subjects was optimized based on accumulated experience from previous analyses of [^11^C]PBR28 data [19]. Radioactivity was measured for 10 s in a well counter cross-calibrated with the PET system. After centrifugation, 0.2 ml plasma was pipetted and plasma radioactivity was measured in a well counter.

Plasma radioactivity corresponding to unchanged [^11^C]PBR28 was determined in arterial blood sampled at 4, 10, 20, 30, 40, 50, 70, and 90 min in patients and at 4, 10, 20, 30, 40, 50 and 72 min in control subjects. The plasma obtained after centrifugation of blood was deproteinized with acetonitrile and analysed by high-performance liquid chromatography [25, 26]. The free fraction of [^11^C]PBR28 in plasma was measured essentially as previously described [27]. The time curve representing the plasma radioactivity of unchanged radioligand was generated based on data obtained from ABSS and manual blood and plasma samples according to previously described procedures [15, 28]. Correction for radioactive metabolites was performed using a population-based approach to fit the fraction of parent radioligand in the plasma using an empirical model consisting of a mixture of the Hill and Richards equations [29].

### PET image analysis

PET images were corrected for head movement using a frame-by-frame realignment algorithm, in which all frames were individually realigned to the first minute of data acquisition. Parametric images of the total distribution volume (*V*_T_) for [^11^C]PBR28 and binding potential (BP_ND_) for [^18^F]FE-PE2I were generated as previously described [15] using the wavelet-aided parametric imaging approach [30] based on a multilinear version of Logan graphical analysis. Parametric images for [^11^C]PBR28 were generated based on 63 min of data acquisition. The parametric images were spatially normalized based on parameters obtained from segmentation and coregistration of MR images. Regions of interest (ROIs) for analysis of [^11^C]PBR28 binding in the cerebellum, limbic cortices and thalamus were defined using the automated anatomical labelling template [31], and ROIs for analysis of radioligand binding in nigrostriatal regions and tracts were defined using a [^18^F]FE-PE2I PET template according to previously described procedures [32]. In addition, the software package FreeSurfer v. 5.0 [33, 34] was applied for parcellation of brain structures for volumetric analysis based on individual MR images.

Differences in regional [^11^C]PBR28 *V*_T_ between the two groups were also examined by voxel-based analysis, using SPM5 (Statistical Parametric Mapping, Wellcome Trust Centre for Neuroimaging). To assess differences in *V*_T_ between controls and PD patients a two-sample *t* test was used, in which the two groups were entered separately and TSPO genotype, age and gender were entered as covariates. As alternative methods to assess the regional binding of [^11^C]PBR28, the averaged radioactivity (expressed as standardized uptake value, SUV) between 40 and 60 min of data acquisition and distribution volume ratios (DVR) for ROIs relative to the cerebellum were calculated as previously described [35].

### Statistical analysis

The difference between control subjects and patients in terms of age was assessed using a *t* test for independent samples. Based on the Kolmogorov-Smirnov and Shapiro-Wilk tests, radiochemistry data (injected radioactivity, injected mass and molar radioactivity) were not considered to be normally distributed. Differences in these parameters between groups were consequently analysed using the Mann-Whitney *U* test. Group differences in regional [^11^C]PBR28 binding (*V*_T_, DVR or SUV) were analysed using two-way ANOVA, with TSPO genotype and diagnostic group as independent variables, and [^11^C]PBR28 binding as the dependent variable. In addition, the strength of evidence in favour of the null hypothesis (no effect of group or genotype) or alternative hypothesis (effect of group or genotype) was estimated from the Bayes factors computed by Bayesian ANOVA. Pearson’s correlation coefficient was used to analyse the associations between *V*_T_ values for [^11^C]PBR28 and BP_ND_ values for [^18^F]FE-PE2I in the putamen and substantia nigra of the more severely affected hemisphere based on clinical ratings. The threshold for statistical significance was set at *P* < 0.05.

## Results

### Demographic and clinical data

**Fig. 1:**
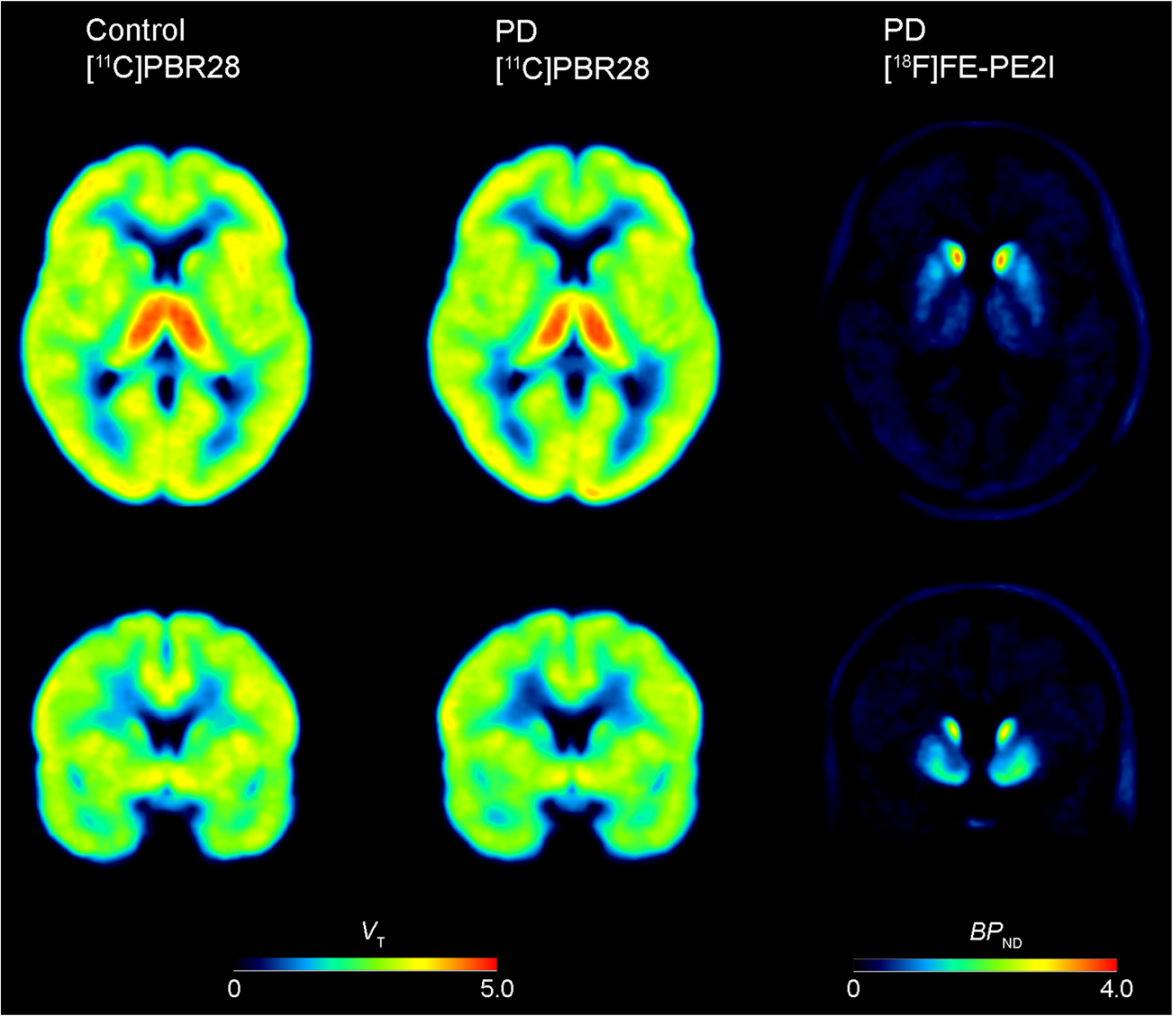
Average parametric images of [^11^C]PBR28 *V*_T_ in control subjects (*left*) and PD patients (*centre*) and of [^18^F]FE-PE2I BP_ND_ in PD patients (*right*). The areas with high [^11^C]PBR28 *V*_T_ represent binding in the thalamus

There was no statistically significant difference in age between control subjects and PD patients (Table 1; *P* = 0.97). Based on genotype analysis of the TSPO rs6971 polymorphism, 16 subjects (8 control subjects and 8 PD patients) were identified as high-affinity binders (HABs) and the remaining 16 subjects were identified as mixed-affinity binders (MABs). In patients, the PET images of [^18^F]FE-PE2I indicated low DAT binding in striatal regions, confirming the diagnosis of PD (Fig. 1). The [^18^F]FE-PE2I BPs for the caudate nucleus (mean 1.6, SD 0.6) and putamen (mean 0.89, SD 0.3) were low relative to the corresponding values for a reference control group (*n* = 20) of the same age (mean 4.0, SD 0.6, for the caudate nucleus; mean 4.4, SD 0.6, for the putamen) [36]. Regional volumes of the brain structures analysed are presented in Supplementary Table S1.

The [^11^C]PBR28 injected radioactivity did not differ significantly between patients and control subjects (*P* = 0.22). Molar radioactivity for [^11^C]PBR28 was higher (*P* = 0.01) and injected mass of the compound was lower (*P* = 0.01) in patients than in control subjects (Table 1). No statistically significant correlation was found between molar radioactivity or injected mass and estimated [^11^C]PBR28 *V*_T_ values in HABs (*P* > 0.13) or MABs (*P* > 0.15; Supplementary Fig. S1).

### Plasma radioactivity data for [^11^C]PBR28

Time curves for the fraction of parent radioligand in plasma indicated a slower rate of metabolism in PD patients, with group differences reaching statistical significance (*P* < 0.05) for samples collected at 30, 40, 50 and 70 min after tracer injection (Supplementary Fig. S2). However, the area under the curve for the metabolite-corrected plasma radioactivity did not differ significantly between control subjects and PD patients (*P* = 0.75; Fig. S3a). Estimates of the free fraction in plasma were higher in patients (mean 0.11, SD 0.020) than in control subjects (mean 0.049, SD 0.020; *P* < 0.001; Fig. S3b).

### [^11^C]PBR28 binding to the TSPO in control subjects and PD patients

**Fig. 2:**
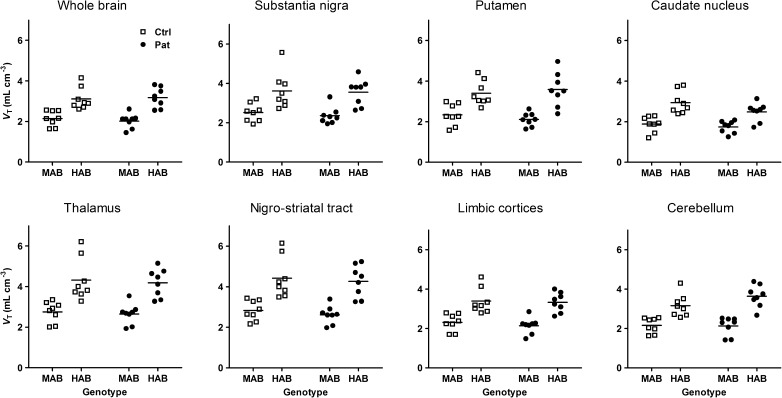
[^11^C]PBR28 *V*_T_ values of selected brain regions in control subjects and PD patients. *MAB* mixed-affinity binder, *HAB* high-affinity binder

Parametric images of the average *V*_T_ for [^11^C]PBR28 in control subjects and PD patients are shown in Fig. 1. Global analysis of *V*_T_ for the whole brain region showed a main effect of genotype (*P* < 0.001; BF_10_ 76,312), but no statistically significant effect of diagnostic group (*P* = 0.86; BF_10_ 0.34), or a group by genotype interaction (*P* = 0.56; BF_10_ 0.50). For all regions studied, the two-way ANOVA revealed a main effect of TSPO genotype with higher [^11^C]PBR28 *V*_T_ in HABs than in MABs (*P* < 0.001; BF_10_ >1,000), but no statistically significant effect of diagnostic group (*P* > 0.05; Fig. 2; Table S2). A Bayesian ANOVA provided evidence in favour of the hypothesis of no difference in *V*_T_ between the groups in the substantia nigra, putamen, nigrostriatal tract, limbic cortices, thalamus and cerebellum (BF_10_ 0.34–0.43). For the caudate nucleus an effect of group at the trend level was found, with lower [^11^C]PBR28 *V*_T_ in patients than in control subjects (*P* = 0.056; BF_10_ 0.68). No statistically significant group by genotype interactions on [^11^C]PBR28 *V*_T_ were found for any of the regions analysed (*P* > 0.15; BF_10_ 0.41–0.88). Voxel-wise analysis of parametric maps for [^11^C]PBR28 *V*_T_ revealed no clusters of statistically significant differences between control subjects and PD patients (results not shown).

Comparison of [^11^C]PBR28 *V*_T_ values obtained using ROI definition in individual space showed a main effect of TSPO genotype (*P* < 0.001; BF_10_ >570), but no statistically significant effect of diagnostic group (*P* > 0.13; BF_10_ 0.34–0.50) or a group by genotype interaction (*P* > 0.13; BF_10_ 0.44–0.92; Table S3). Analysis of the average SUV for the 40–60 min data acquisition (Table S4) showed a main effect of genotype (*P* < 0.001; BF_10_ 113–2,168), but no statistically significant effect of diagnostic group (*P* > 0.23; BF_10_ 0.34–0.45), or a group by genotype interaction (*P* > 0.27; BF_10_ 0.40–0.62).

Analysis of the ratio of *V*_T_ for regions of interest relative to that for the cerebellum (DVR) revealed no statistically significant main effect of TSPO genotype for any of the regions studied (*P* > 0.05; BF_10_ 0.34–0.93; Fig. S4). For the caudate nucleus, the nigrostriatal tract, limbic cortices and thalamus there were statistically significant effects of diagnostic group with lower DVR in PD patients than in controls (*P* < 0.05; BF_10_ 2.4–13). A statistically significant group by genotype interaction on DVR was found for the caudate nucleus (*P* = 0.013; BF_10_ 4.2), but not for other regions analysed (*P* > 0.05; BF_10_ 0.47–1.7).

### Correlation between binding to DAT and to TSPO in PD patients

**Fig. 3:**
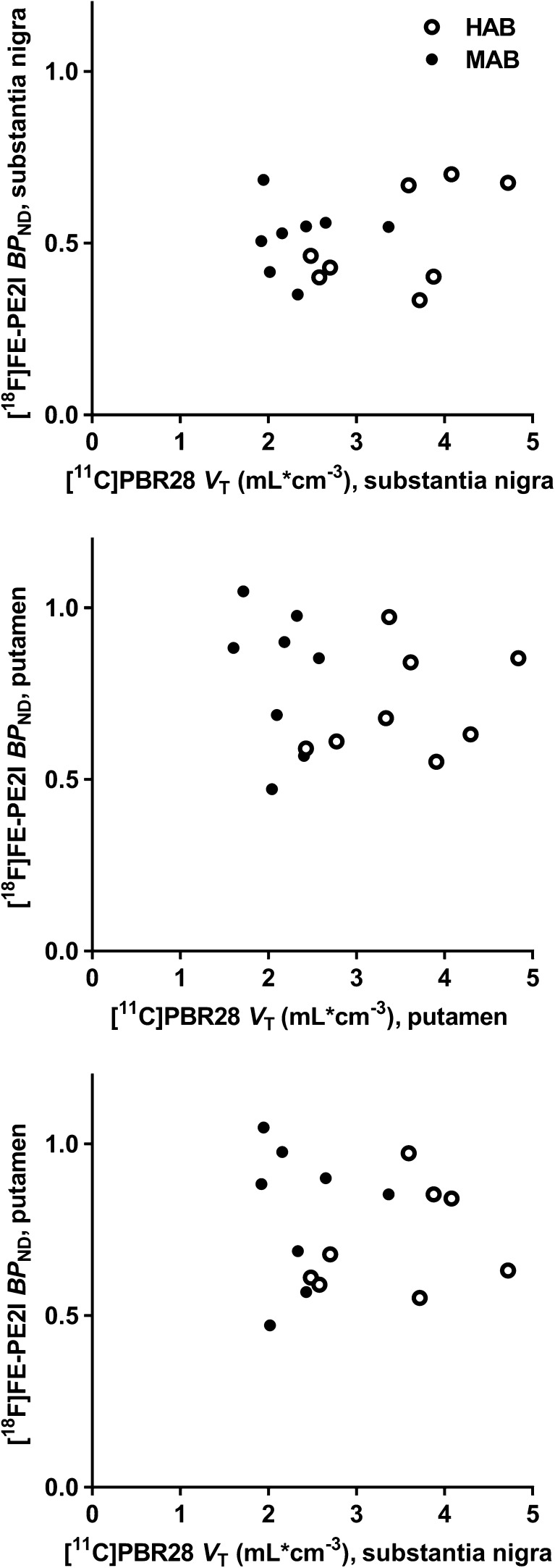
Correlations between [^11^C]PBR28 *V*_T_ and [^18^F]FE-PE2I BP_ND_ in PD patients for the substantia nigra (*top*) and putamen (*centre*), and between [^18^F]FE-PE2I BP_ND_ for the putamen and [^11^C]PBR28 *V*_T_ for the substantia nigra (*bottom*). *MAB* mixed-affinity binder, *HAB* high-affinity binder

Analysing data from all patients, *V*_T_ values for [^11^C]PBR28 did not correlate with [^18^F]FE-PE2I BP_ND_ values for binding to DAT in the putamen (*r* = −0.14, *P* = 0.62) or the substantia nigra (*r* = 0.28, *P* = 0.29; Fig. 3). Analysing data from HABs and MABs separately, no statistically significant correlations were found between binding to DAT and to TSPO: in HABs, *r* = 0.30, *P* = 0.48, in the putamen, and *r* = 0.56, *P* = 0.15, in the substantia nigra; in MABs, *r* = −0.24, *P* = 0.56, in the putamen, and *r* = 0.050, *P* = 0.91, in the substantia nigra. With regard to correlations between TSPO binding in the substantia nigra and DAT binding in the putamen, no statistically significant correlations were found when analysing data from all patients (*r* = −0.044, *P* = 0.87), or when analysing data from HABs and MABs separately (in HABs, *r* = 0.30, *P* = 0.46; in MABs, *r* = 0.0088, *P* = 0.98; Fig. 3).

## Discussion

Neuroinflammatory processes, involving expression of activated microglia, are considered to be of key relevance for processes underlying brain pathology in PD; however, previous PET studies of the microglial TSPO protein in PD have yielded inconsistent results. In the present study 16 PD patients and 16 matched control subjects were examined using PET and the second generation TSPO radioligand [^11^C]PBR28 as a marker of microglial activation. The results confirmed the well-known effect of TSPO genotype on [^11^C]PBR28 binding, whereas no evidence was found for a difference in TSPO binding between PD patients and control subjects, or for a correlation between the binding to TSPO and DAT in PD patients.

The results corroborate previous findings with the second generation TSPO radioligand [^18^F]FEPPA [13, 14], but are inconsistent with results showing elevated TSPO binding using the radioligands [^11^C]PK11195 [7–10] or [^11^C]DPA713 [12] to study brain TSPO in PD. PBR28 has been found to display two distinct affinity sites, including a high-affinity binding site (*K*_i_ 3.4 nM) and a low-affinity binding site (*K*_i_ 188 nM [37]), a feature also shared by [^18^F]FEPPA [38]. Such a bimodal binding pattern has not been observed with [^11^C]PK11195 and is less evident for DPA713, which displays a difference of about fourfold in affinity for high-affinity than for low-affinity binding sites [37]. Affinity of PBR28 for the high-affinity binding site has been found to be higher than that of PK11195 (*K*_i_ 28 nM) and DPA713 (*K*_i_ 15 nM), confirming a superior signal-to-noise ratio for detecting group differences in TSPO binding to the high-affinity binding site with [^11^C]PBR28 than with the latter tracers. However, owing to low affinity for low-affinity binding sites, binding to these sites cannot be quantified using second generation radioligands, such as [^11^C]PBR28 and [^18^F]FEPPA. Thus, it cannot be excluded that findings of studies using [^11^C]PK11195 as a radioligand reflect the PBR28 low-affinity binding site.

Given the progressive neurodegenerative nature of PD, inflammatory processes are expected to vary during the course of the disease. It may thus be suggested that the discrepant findings of the current and earlier investigations could reflect differences in disease severity between the groups of patients recruited for these studies. However, differences in disease severity are unlikely to explain the inconsistency in the findings of previous investigations, in which increased TSPO binding was found in patients examined during the early stage [8, 9] or later stages [7, 10, 12] of PD, and the findings of the more recent studies, in which increases in TSPO binding were not observed in PD patients with various disease durations and severities [13, 14]. Nevertheless, it cannot be excluded that clinical or neurochemical heterogeneity related to the disease course between the groups of patients examined may account for the inconsistent findings of TSPO PET studies in PD.

Another possible explanation for the discrepant findings is the difference in methods for quantitative evaluation of brain TSPO employed in the studies. The present findings, similar to previously reported results [13, 14], rely on the work-demanding use of the arterial plasma concentration as an input function, whereas the other studies are based on cluster analysis for definition of nondisplaceable radioligand binding. Because of the different methodology for PET data quantification and outcome measures (*V*_T_ or BP_ND_) used for the assessment of TSPO binding, the findings cannot be directly compared.

Our findings obtained using [^18^F]FE-PE2I as a radioligand show a marked reduction in nigrostriatal binding to the DAT in the PD patients examined. However, despite the marked reduction in dopaminergic innervation, no evidence was found for a change in brain TSPO binding in the same patients. The findings of the two earlier PET studies combining imaging of TSPO using [^11^C]PK11195 as a radioligand and dopaminergic markers in PD were inconclusive. An inverse correlation between midbrain TSPO binding and dopaminergic innervation in the putamen was found in a group of drug-naive patients with early PD [8], but this finding was not confirmed in a group of more severely affected patients [7]. Taken together, the findings suggest that increased TSPO binding may parallel dopaminergic pathology during the early stages of the disease, but does not seem to be a marker of ongoing dopaminergic degeneration in PD.

Plasma protein binding measurements indicated differences in the estimates of the free fraction of [^11^C]PBR28 between PD patients and control subjects. Given that differences in the plasma free fraction would be expected to yield biased estimates of group differences in cerebral TSPO binding when using *V*_T_ as an outcome measure, correction of *V*_T_ for the plasma free fraction has been suggested as a more suitable approach to the quantitative analysis of [^11^C]PBR28 data [39]. Accuracy in the determination of protein binding is limited by the sensitivity of the detection methods employed. However, the reliability of the estimates of plasma protein binding has been reported to be poor for [^11^C]PBR28 [19]. A correction for the free fraction of radioligand in plasma was consequently not applied in the present study.

To overcome methodological limitations in the measurement of plasma radioactivity, the use of ratio-based reference tissue methods has been proposed as an alternative for quantitative analysis of [^11^C]PBR28 PET data [35]. In the present study the DVR relative to the cerebellum was used as an alternative outcome measure. Using this method, regional DVR values were found to be statistically significantly lower in PD patients than in controls. In a recent test–retest study, DVR showed poor reliability and validity as an outcome measure for the quantitative assessment of cerebral [^11^C]PBR28 binding [40]. Because regional *V*_T_ values are highly correlated, normalizing these values by *V*_T_ for another region results in low residual variability and consequently high sensitivity to noise [40]. For this reason *V*_T_ was considered as the preferred outcome measure for quantification of [^11^C]PBR28 PET data in the present study.

PD patients and control subjects were examined in two studies conducted approximately 3 years apart. Methodological factors that could have varied between the two series should be considered as possible limitations of the investigation. Molar radioactivity for [^11^C]PBR28 was found to be higher in PD patients than in controls, resulting in a higher injected mass in control subjects. However, the low masses of PBR28 injected (0.2–1.5 μg, 7–53 pmol/kg) would be expected to have had only a minor impact on ^11^C-PBR28 *V*_T_ values. In support of this notion, no evident correlation was found between *V*_T_ values and molar radioactivity or injected mass of PBR28. In addition, based on examples with other tracers with nanomolar affinities for the target protein, such low masses have been predicted to induce less than 1% target occupancy when administered to human subjects [41, 42]. For these reasons, the impact of the mass of PBR28 is considered to have been negligible when comparing *V*_T_ values between PD patients and controls.

The use of different MR scanners with magnetic field strengths of 1.5 T and 3 T in patients and control subjects, respectively, is another possible limitation of the study. To minimize the impact of MR image quality on PET data quantification, ROIs were defined in a standardized space, thus limiting the use of information from individual MR images to the spatial normalization of PET images. While different MR acquisition procedures are unlikely to have confounded the results of the PET quantitative analysis, the group comparison of brain morphometry data (Table S1) should be interpreted with caution, as differences in magnetic field strength may have introduced bias in the estimates of regional brain volumes obtained by the methodology employed [43]. A possible influence of partial volume effects resulting from a difference between groups in grey matter volumes cannot be excluded, although the homogeneous binding of [^11^C]PBR28 across regions suggests that the impact of partial volume effects was minimal.

With the limitations inherent to the quantification of TSPO PET data, in vitro studies of post-mortem tissue allowing direct measurement of protein levels in the brain are of critical importance for interpreting findings obtained in vivo. Although in vitro studies have provided evidence for microglial activation in PD [2, 3], investigation of TSPO radioligand binding is to our knowledge limited to a study of tissue samples from three PD patients [44] that showed a marked increase, albeit with high within-group variability. Thus, the field would benefit from large-scale post-mortem investigations further addressing the topic of TSPO binding in PD.

While TSPO imaging is widely applied as a noninvasive marker of neuroinflammation, TSPO is not an ideal target for assessment of inflammatory processes. This protein is known to be expressed by multiple cell types other than activated microglia [6]. Moreover, endogenous ligands including cholesterol and porphyrins [45, 46] display high affinity for this protein. Such factors could account for the interindividual variability and discrepant findings observed in TSPO PET studies. Future development of tracers specifically targeting microglia activation is required to elucidate the role of inflammatory processes in neurodegeneration.

In conclusion, the present findings obtained using [^11^C]PBR28 PET do not support the hypothesis of elevated cerebral TSPO binding, or a relationship between TSPO binding and nigrostriatal degeneration, in PD. The results are consistent with previous findings obtained using the second generation radioligand [^18^F]FEPPA.

## Electronic supplementary material

ESM 1 (PDF 875 kb)
