# Supplementary material for: PET imaging of [11C]PBR28 in Parkinson’s disease patients does not indicate increased binding to TSPO despite reduced dopamine transporter binding
Source: Eur J Nucl Med Mol Imaging. 2018 Oct 1;46(2):367–75. doi: 10.1007/s00259-018-4161-6 (PMC6333720; doi:10.1007/s00259-018-4161-6)
Supplement: Supplementary file 1 — (PDF 875 kb) [file 259_2018_4161_MOESM1_ESM.pdf]

Supplementary Information

PET imaging of [^{11}C]PBR28 in Parkinson's disease patients does not indicate increased binding to TSPO despite reduced dopamine transporter binding

European Journal of Nuclear Medicine and Molecular Imaging

Authors: Katarina Varnäs, Zsolt Cselényi, Aurelija Jucaite, Christer Halldin, Per Svenningsson, Lars Farde, Andrea Varrone

Correspondence: Katarina Varnäs, Karolinska Institutet, Department of Clinical Neuroscience, Centre for Psychiatry Research, Karolinska University Hospital, Stockholm, Sweden
Email: katarina.varnas@ki.se

Supplementary Tables

Table S1 Volumes of the brain structures analysed. Volumes of the brain regions were estimated using the software package FreeSurfer

	Volume, mean (SD)	
Brain region	Control subjects (*n*=16)	PD patients (*n*=16)
Intracranial volume (mL)	1657 (159)	1843 (111)
Relative volumes *		
Caudate nucleus	0.42 (0.042)	0.41 (0.039)
Putamen	0.59 (0.047)	0.58 (0.076)
Limbic cortices	2.3 (0.17)	2.0 (0.20)
Thalamus	0.83 (0.081)	0.91 (0.063)
Cerebellum	7.0 (0.70)	7.2 (0.56)

* Absolute volume divided by intracranial volume and multiplied by 100.

Table S2 V_T values for [^{11}C]PBR28 for the different diagnostic and genotype groups obtained using ROI definitions in normalized space. Values are presented as mean (SD). MAB, Mixed-affinity binder; HAB, high-affinity binder

	Control subjects			PD patients		
Brain region	MAB	HAB	Difference (%)	MAB	HAB	Difference (%)
Whole brain	2.1 (0.38)	3.1 (0.55)	31	2.0 (0.36)	3.2 (0.49)	36
Substantia nigra	2.5 (0.45)	3.6 (0.92)	30	2.4 (0.44)	3.6 (0.68)	34
Caudate nucleus	1.9 (0.39)	2.9 (0.56)	36	1.7 (0.30)	2.5 (0.45)	30
Putamen	2.3 (0.53)	3.4 (0.60)	31	2.1 (0.33)	3.6 (0.83)	41
Nigro-striatal tract	2.8 (0.49)	4.4 (0.99)	36	2.6 (0.44)	4.3 (0.78)	39
Limbic cortices	2.3 (0.43)	3.4 (0.64)	32	2.1 (0.41)	3.3 (0.49)	36
Thalamus	2.8 (0.51)	4.3 (1.0)	36	2.6 (0.50)	4.2 (0.69)	37
Cerebellum	2.2 (0.38)	3.2 (0.57)	32	2.1 (0.46)	3.6 (0.56)	42

Table S3 V_T values, mean (SD) for [^{11}C]PBR28 obtained using ROI definitions in individual space. MAB, Mixed-affinity binder; HAB, high-affinity binder

	Control subjects			PD patients		
Brain region	MAB	HAB	Difference (%)	MAB	HAB	Difference (%)
Whole brain	2.2 (0.39)	3.1 (0.54)	31	2.0 (0.35)	3.2 (0.53)	37
Substantia nigra	2.5 (0.42)	3.5 (0.94)	28	2.3 (0.44)	3.5 (0.67)	34
Caudate nucleus	1.9 (0.40)	2.9 (0.51)	35	1.8 (0.32)	2.6 (0.47)	33
Putamen	2.3 (0.50)	3.4 (0.61)	33	2.1 (0.32)	3.5 (0.74)	41
Nigro-striatal tract	2.8 (0.51)	4.4 (0.83)	35	2.8 (0.44)	4.7 (1.1)	42
Limbic cortices	2.4 (0.45)	3.4 (0.64)	31	2.2 (0.41)	3.4 (0.56)	36
Thalamus	2.8 (0.52)	4.3 (0.92)	35	2.7 (0.49)	4.5 (0.92)	40
Cerebellum	2.2 (0.42)	3.2 (0.57)	32	2.1 (0.43)	3.7 (0.67)	43

Table S4 Average standardized uptake values for [^{11}C]PBR28 radioactivity concentration between 40 and 60 minutes after injection. Mean (SD) for diagnostic and genotype groups. MAB, Mixed-affinity binder; HAB, high-affinity binder

	Control subjects			PD patients		
Brain region	MAB	HAB	Difference (%)	MAB	HAB	Difference (%)
Whole brain	0.81 (0.12)	1.1 (0.23)	28	0.84 (0.14)	1.1 (0.20)	25
Substantia nigra	0.97 (0.16)	1.3 (0.32)	25	1.0 (0.20)	1.4 (0.22)	26
Caudate nucleus	0.71 (0.14)	1.1 (0.22)	34	0.75 (0.16)	1.0 (0.18)	23
Putamen	0.84 (0.14)	1.2 (0.25)	30	0.85 (0.16)	1.2 (0.26)	30
Nigro-striatal tract	1.0 (0.15)	1.5 (0.29)	30	1.1 (0.16)	1.6 (0.32)	30
Limbic cortices	0.87 (0.13)	1.2 (0.26)	29	0.89 (0.16)	1.2 (0.20)	25
Thalamus	1.0 (0.18)	1.5 (0.31)	29	1.1 (0.20)	1.5 (0.29)	27
Cerebellum	0.78 (0.16)	1.1 (0.27)	30	0.82 (0.17)	1.2 (0.25)	34

Supplementary Figures

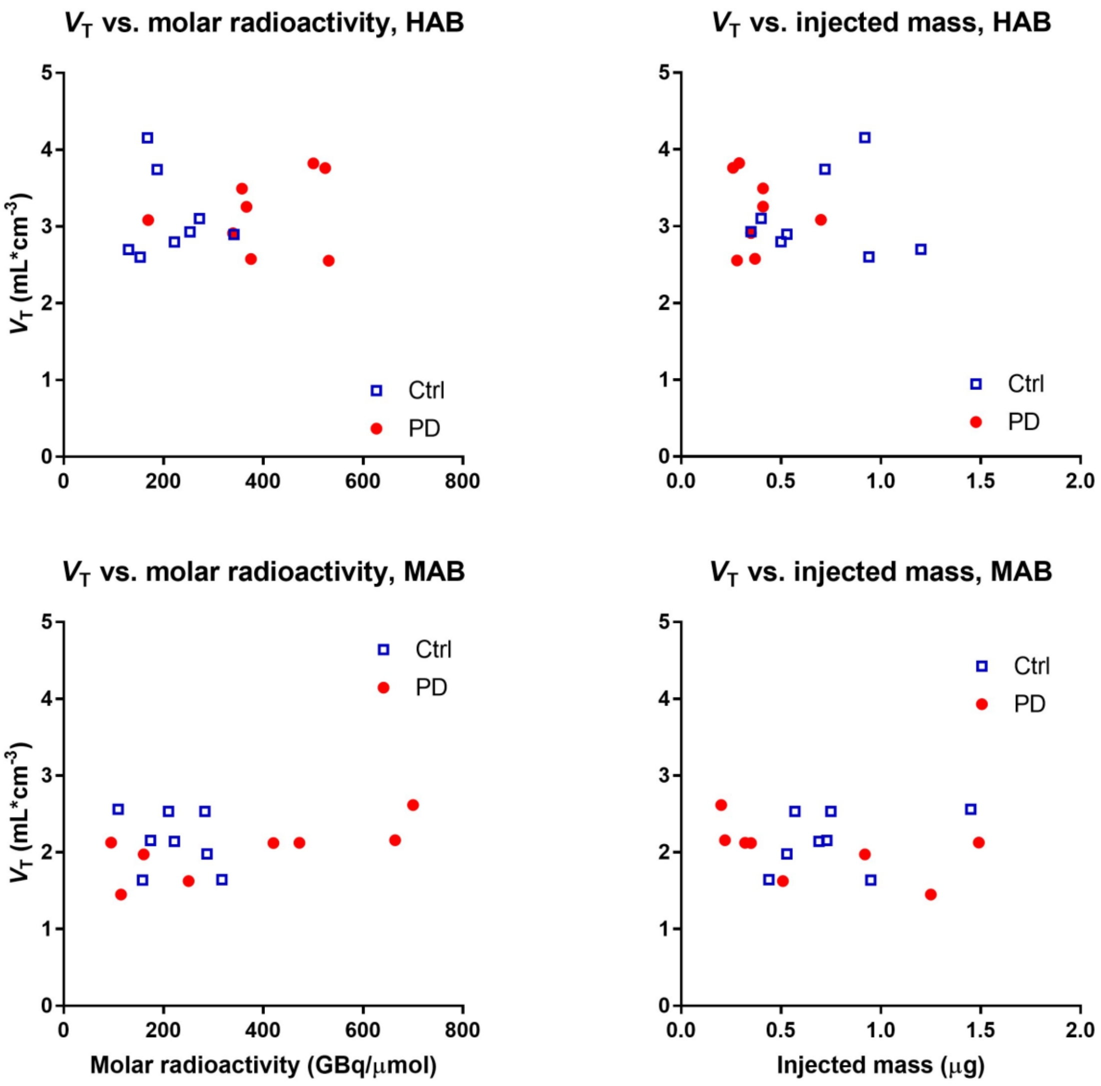

Figure S1 Relationship between [11C]PBR28 whole brain V_T values and molar radioactivity (left), and injected mass (right) for high-affinity binders (HAB; upper panel) and mixed-affinity binders (MAB; lower panel)

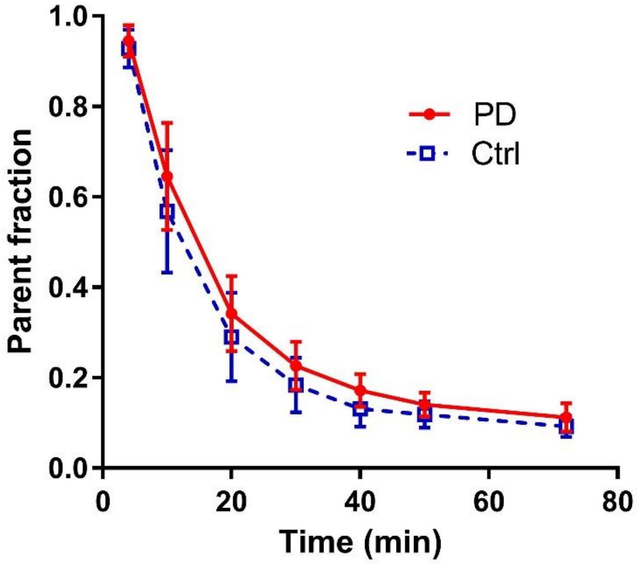

Figure S2 Plasma radiometabolism for [^{11}C]PBR28 in control subjects and PD patients. Data are presented as mean (SD) of the fraction of unchanged radioligand in plasma

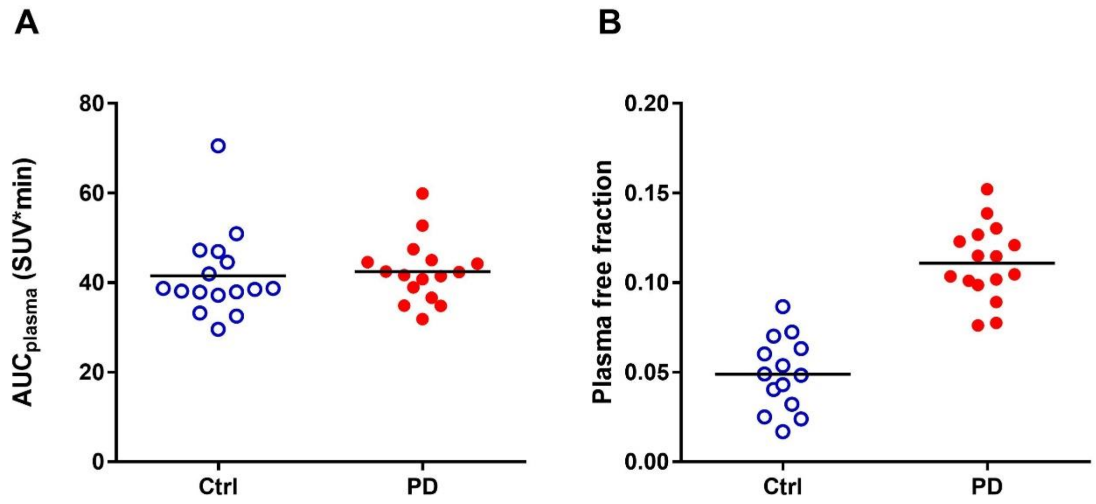

Figure S3 Area under the curve for plasma radioactivity (A) and the free fraction of radioligand in plasma (B) for [^{11}C]PBR28 in control subjects and PD patients

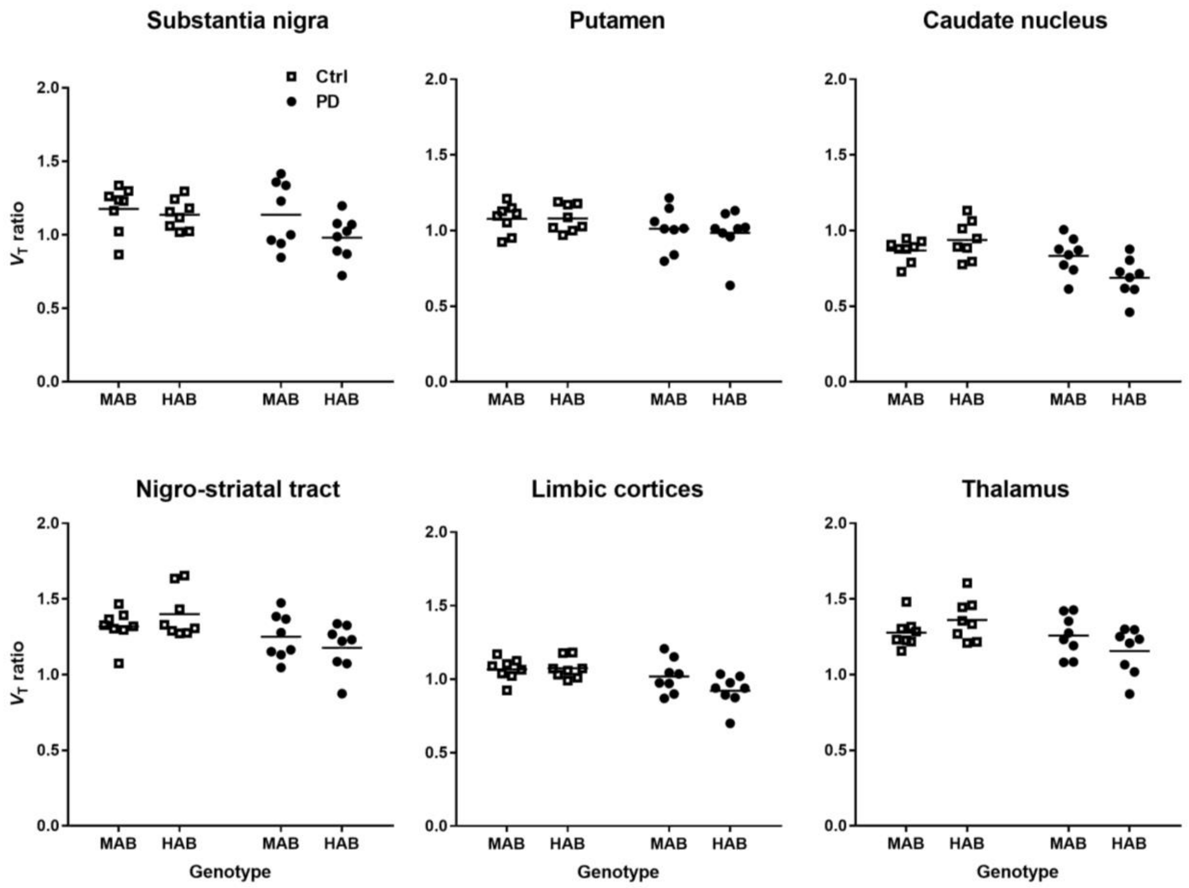

Figure S4 [^{11}C]PBR28 distribution volume ratio for regions of interest relative to the cerebellum for control subjects and PD patients
